# *In Vitro* HIV-1 Evolution in Response to Triple Reverse Transcriptase Inhibitors & *In Silico* Phenotypic Analysis

**DOI:** 10.1371/journal.pone.0061102

**Published:** 2013-04-17

**Authors:** Barbara A. Rath, Kaveh Pouran Yousef, David K. Katzenstein, Robert W. Shafer, Christof Schütte, Max von Kleist, Thomas C. Merigan

**Affiliations:** 1 Department of Pediatrics, Division of Pneumonology-Immunology, Charité University Medical Center, Berlin, Germany; 2 Department of Mathematics and Computer Science, Free University, Berlin, Germany; 3 Center for AIDS Research, Stanford University Medical Center, Stanford, California, United States of America; McGill University AIDS Centre, Canada

## Abstract

**Background:**

Effectiveness of ART regimens strongly depends upon complex interactions between the selective pressure of drugs and the evolution of mutations that allow or restrict drug resistance.

**Methods:**

Four clinical isolates from NRTI-exposed, NNRTI-naive subjects were passaged in increasing concentrations of NVP in combination with 1 µM 3 TC and 2 µM ADV to assess selective pressures of multi-drug treatment. A novel parameter inference procedure, based on a stochastic viral growth model, was used to estimate phenotypic resistance and fitness from *in vitro* combination passage experiments.

**Results:**

Newly developed mathematical methods estimated key phenotypic parameters of mutations arising through selective pressure exerted by 3 TC and NVP. Concentrations of 1 µM 3 TC maintained the M184V mutation, which was associated with intrinsic fitness deficits. Increasing NVP concentrations selected major NNRTI resistance mutations. The evolutionary pathway of NVP resistance was highly dependent on the viral genetic background, epistasis as well as stochasticity. Parameter estimation indicated that the previously unrecognized mutation L228Q was associated with NVP resistance in some isolates.

**Conclusion:**

Serial passage of viruses in the presence of multiple drugs may resemble the selection of mutations observed among treated individuals and populations *in vivo* and indicate evolutionary preferences and restrictions. Phenotypic resistance estimated here “*in silico*” from *in vitro* passage experiments agreed well with previous knowledge, suggesting that the unique combination of “wet-” and “dry-lab” experimentation may improve our understanding of HIV-1 resistance evolution in the future.

## Introduction

Antiretroviral drug resistance [1] limits therapeutic options, clinical benefit and increases the risk of clinical progression [2]. Recommended first-line ART regimens consist of one non-nucleoside reverse transcriptase inhibitor (NNRTI) combined with two nucleoside reverse transcriptase inhibitors (NRTIs) [3]. All drugs within these combinations exert their effect on the HIV-1 reverse transcriptase. Nevirapine (NVP), the first approved NNRTI, binds directly to reverse transcriptase (RT) (the NNRTI binding pocket), leading to conformational inflexibility [4] and inhibition of enzymatic action [5]. NVP is used frequently to prevent the transmission of HIV-1 from mother to child [6]. Lamivudine (3 TC) is the most commonly used NRTI. Its triphosphate (3 TC-TP) competes with endogenous deoxycytosine triphosphate for incorporation into the nascent viral DNA during reverse transcription, where it inhibits HIV DNA elongation [7]. Adefovir (ADV) is an adenosine-monophosphate analogue, which in diphosphate form, acts as a chain-terminator competing with deoxyadenosine triphosphate for incorporation into viral DNA. Although not approved by the FDA for treatment of HIV [8], it is closely related to tenofovir disoproxil fumarate (TDF) a preferred nucleotide RT inhibitor that is currently recommended as a key component in first-line HAART [9].

Resistance to NVP, 3 TC and ADV is attributed to distinct mutations. NVP resistance mutations within the NNRTI binding pocket decrease NVP binding to RT by means of steric hindrance [10]. Lamivudine (3 TC) resistance conferred by the M184V mutation, decreases the affinity of 3 TC-TP for the primer/template complex during reverse transcription [7]. In contrast, ADV (and tenofovir) resistance selectively decreases incorporation of ADV phosphonate into viral DNA [11], [12], associated with mutations at K70E and K65R.

Different mutational trajectories may arise during combination therapy, which may be altered by pre-existing mutations through epistatic constraints and genetic bottlenecks [13], [14]. In the context of combination therapy, selective pressures drive evolutionary pathways, consideration of which may optimize strategic sequencing of ART regimens [1], [15]. Furthermore, the preservation of mutations that limit viral fitness and replicative capacity [16], [17] provide for significant improvement in clinical and immunological outcomes among drug-experienced patients [18].

To understand drug resistance during combination antiviral drug treatments, an *in vitro* assay [19] was established in stimulated PBMC infected with virus isolates from 4 NRTI-experienced (but NNRTI-naive) patients. Mutations were selected by passage in different combinations and concentrations of ADV, 3 TC and NVP and viral fitness and resistance were estimated on the basis of a stochastic model of viral growth.

## Materials

### HIV Strains

As described previously [19], clinical isolates were derived from frozen samples. The primary clinical isolates were derived from 4 individuals who had previously received NRTI and protease inhibitors, but who had never been exposed to NNRTIs. The baseline RT mutations (as compared to the Los Alamos consensus Hxb2) up to RT amino acid position 300 can be found in Table 1.

**Table 1:** Baseline amino acid substitutions in relation to reference sequence (Hxb2) from the Stanford HIVDB [28].

	Reverse Transcriptase Amino Acid Position																														
	20	35	41	67	69	70	83	90	118	122	123	135	169	177	184	196	201	202	208	210	211	214	215	219	272	275	277	291	293	294	297
Hxb2	K	V	M	D	T	K	R	V	V	E	D	I	E	D	M	G	K	I	H	L	R	F	T	K	A	K	K	E	I	P	E
Iso#1		**T**	**L**					**I**		**K**	**E**			**E**	**V**	**E**	**V**						**Y**			**Q**	**R**		**V**		**Q**
Iso#2	**R**			**S**	**N**	**R**			**I**		**S**	**T**			**V**			**V**				**L**	**F**	**Q**	**P**			**D**			**T**
Iso#3	**R**			**S**	**N**	**R**			**I**		**S**	**T**			**V**			**V**				**L**	**F**	**Q**	**P**			**D**			**T**
Iso#4		**I**	**L**	**N**			**K**			**K**			**D**		**V**				**Y**	**W**	**K**		**Y**						**V**		
Iso#5			**L**							**K**	**E**	**T**			**V**					**W**	**K**		**Y**		**P**					**T**	

### Cells and Cell Culture

HIV-negative donor PBMC from the Stanford Blood Bank were cultured in RPMI medium containing 15% heat-inactivated fetal calf serum, IL-2, PenStrep, and L-Glu and stimulated for 2–3 days with phytohemagglutinin (Sigma, St. Luis, MO) [20]–[22].

### Test Compounds

Nevirapine (NVP) was kindly provided by Boehringer Ingelheim (Ridgefield, CT), lamivudine (3 TC) by GlaxoSmithKline (Research Triangle Park, NC), and adefovir (ADV) by Gilead (Foster City, CA).

## Virological Methods

### Passage Experiments

**Figure 1:**
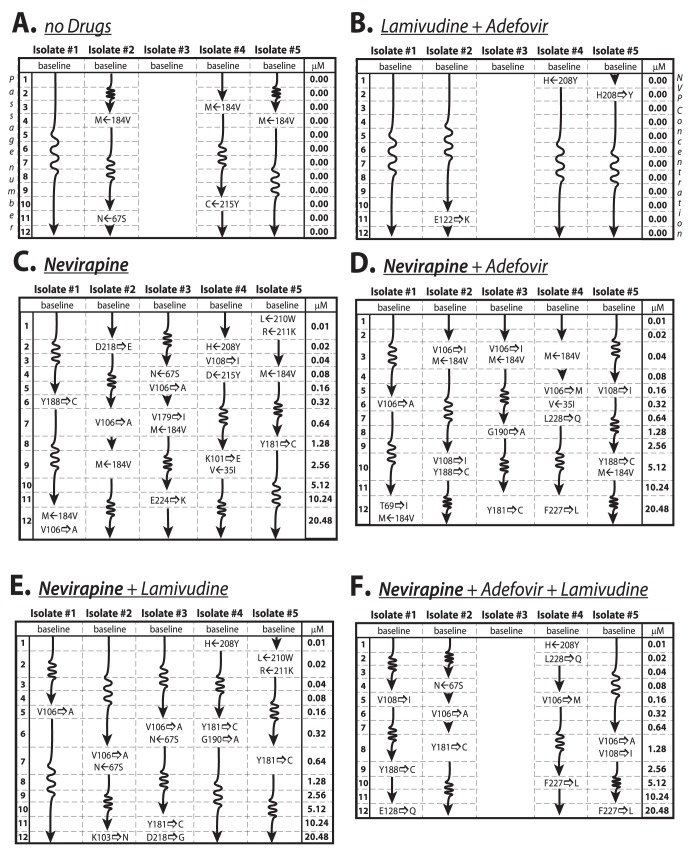
Summary of passage experiments with sequencing data. The illustration provides a complete review of RT sequence changes under the following experimental set-ups: A: no drugs were added to the media, B: [1 µM] 3 TC and [2 µM] ADV were added and maintained, C: NVP was added and concentrations were doubled for each passage (0.01 µM NVP during the first passage up to 20.48 µM during the last passage), D: [2 µM] ADV and increasing concentrations of NVP were added, E: [1 µM] 3 TC and increasing concentrations of NVP were added and F: [1 µM] 3 TC and [2 µM] ADV and increasing concentrations of NVP were added to the medium. Individual isolates #1 to #5 are indicated above the columns. Sequence changes listed are indicated in the rows that correspond to the passage number where they were first observed. NVP concentrations used in the respective passage experiment are listed on the right in units µM. Any mutation away from wild-type (Hxb2 strain) is indicated by a rightward-pointing arrow, whereas reversal to wild-type is indicated by a left-ward pointing arrow. All sequence changes (novel mutations and reversals) persisted throughout passage 12.

The serial combination passage experiments with isolates #1–#5 were conducted as follows [19]: (A) no drugs were added to the media, (B) 1 µM 3 TC and 2 µM ADV were added and maintained, (C) NVP was added and concentrations were doubled with each passage (0.01 µM NVP during the first passage up to 20.48 µM during the last passage), (D) 2 µM ADV and increasing concentrations of NVP were added, (E) 1 µM 3 TC and increasing concentrations of NVP were added and (F) 1 µM 3 TC and 2 µM ADV and increasing concentrations of NVP were added to the medium, see Figure 1. Isolates #2 and #3 were derived from the same individual but were run independently in experimental set-ups C, D & E. For each experimental set-up (A–F), 12 single-passage experiments were run, in total 5* 3* 12+4* 3* 12 = 324 single-passage experiments with a median duration of 21 days, respectively. With each passage-experiment in C, D, E and F, the concentration of NVP was doubled. The NVP starting dose was 0.01 µM, below the previously reported IC_50_ of NNRTI-naïve isolates (around 0.1 µM [23]). The final concentration was 2048-fold (20.48 µM), below reported cytotoxic levels [23]. Cultures were passaged as previously described [19]. Viral growth was monitored using a p24 antigen assay (Abbott Laboratories, Chicago, IL). At p24 ELISA values≥3×10^4^ pg/ml, the cultures were passaged: at levels <3×10^4^ pg/ml, cultures were split, and 2.5 million PBMCs were replaced by new donor PBMC in media containing the respective drugs in the same molar concentration. Supernatant to infect new cells was adjusted by p24 values obtained prior to passage.

### ABI Sequencing

Population-based sequencing of amplified cDNA from viral RNA was performed as described previously [19], [24]. cDNA was obtained using Superscript-One-Step RT-PCR reagent (Life Technologies, Gaithersburg, MD). First-round nested PCR primers were RT-21 [25] and MAW-26 [24], second-round primers were PRO-1 [26] and RT-20 [25]. A d-Rhodamine labeled terminator kit (PE Applied Biosystems, Warrensburg, UK) and the previously described primers RT-a, RT-b (forward), RT-y and HXBR2-89 (reverse) [27] were used for sequencing (ABI Model 377 equipment and software). After alignment, proofreading, and editing, sequence data were compared to baseline and earlier passages of virus. Any change relative to wild type Hxb2 [28] sequence was defined as *mutation*. Any mutation back towards Hxb2 was defined as a *reversal*, even if it was not “all the way back”.

## Mathematical Methods

Novel mathematical methods were developed in order to quantitatively estimate key phenotypic attributes from the experimentally observed viral growth kinetics, by minimizing the residual error ε between experimental- and model-predicted virus *passage times*. The estimated phenotypic attributes include the fold resistance towards NVP, 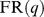, and the fitness deficits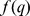 for mutational events 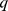 occurring during the respective passage experiments. Furthermore, the growth rate of the respective baseline isolate 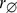, its susceptibility towards NVP (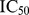) and the probability 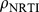 to encounter inhibition by NRTIs (ADV or 3 TC) with intensity 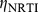 (explained below) were estimated.

The viral growth model is introduced below. Based on the viral growth model, *passage times* were computed to derive an objective function suited for parameter estimation from the available experimental data. Finally, a large-scale model selection technique was used to find the most informative/relevant set of phenotypic parameters and the robustness of the parameter estimation procedure was assessed. The source code for the developed methods is provided in the Material S1, with a short description of the code.

### Basic Viral Growth Model

We assumed a simple-birth Markov model [29], combining the intermediate steps of target cell infection, pro-viral integration, virus release, and virus maturation (e.g. see [30] for an overview). Target cell concentrations were held constant during the experiments (see *Virological Methods*), which reduces the infection to first order kinetics. Furthermore, the absence of immune responses *in vitro* allows the assessment of virus growth in the absence of the immunologic confounders typically encountered *in vivo*. For each virus isolate we could therefore model viral growth kinetics with rate constant 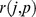, where the index *j* refers to the experiment 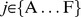 and the index *p* refers to the passage number, i.e. passage 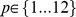, as shown in Fig. 1. For example, isolate #1 is assumed to grow at rate 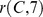
in experiment C at passage 7, i.e. in the presence of 0.32 µM NVP and after having acquired mutation Y188
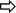
C (see Fig. 1C). As explained in the example, the viral growth with rate constant 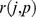 is determined by the presence of baseline mutations, by the presence of drugs at different concentrations, and by mutational events 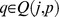, arising throughout the course of the experiment (selection/de-selection of mutations). We modelled their simultaneous effects as described previously [31]:
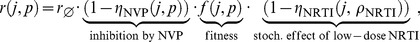
(1)where 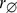 denotes the growth rate of the baseline viral isolate in the absence of drugs. The parameter 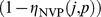 denotes the **effect of NVP** on viral growth kinetics in experiment *j*, in passage *p*. It holds that 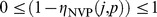, i.e. when the drug is very efficient, 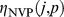 will be close to 1 (computation outlined below). The fitness deficit of the viral strain encountered in experiment *j*, passage *p* is denoted by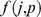. If the strain is very fit, 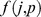 will be close to 1, if it is very unfit, it will be close to 0 (computation outlined below). The parameter 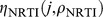 denotes the intensity of the **NRTI**-**effect** (ADV/3TC effects) on viral growth in experimental set-up *j*, which was estimated to be 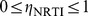 with probability 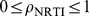 when NRTIs were added (experimental set-ups 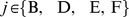) and which was set to 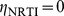, if NRTIs were not added (experimental set-up: 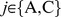). Note, that the concentrations of NRTIs were maintained throughout an experimental set-up *j*, in contrast to NVP concentrations, which were increased in consecutive passages *p*. Different models for ADV/3TC effects were compared: (i) assigning individual “noise effects” to ADV and 3TC respectively, (ii) assigning an isolated ADV effect or (iii) a 3TC effect versus (iv) a model that assigned an NRTI-effect if one of the inhibitors was present. The comparison suggested that model (iv) was best suited to describe the data. We therefore assigned an NRTI**-**effect, if at least one of the inhibitors (ADV or 3TC) was present in experimental set-up *j*.

### Drug Effects and Fitness

The **effect of NVP** on viral growth was modelled according to the standard model of pharmacological action (Emax-model) [32]:
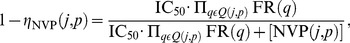
(2)where 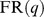 denotes the fold resistance to NVP exerted by a single mutation 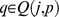 selected during the course of experiment *j* until passage *p*. [NVP (*j*, *p*)] denotes the concentration of NVP added in passage *p* of experiment 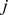 and 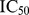 refers to the fifty percent inhibitory concentration of the baseline isolate.

Accordingly, the fitness deficit 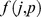 of the respective viral strain present at passage p during experiment j was modelled according to:
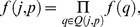
(3) where 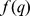 denotes the relative fitness of the single mutation 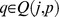, that has not yet been reversed/de-selected until passage p in experiment j. Note, that all mutational events 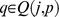 that have arisen until a particular passage 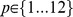 in experiments 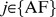 were taken into account simultaneously. For example, in experimental set-up*j* = A for isolate #2, at passage *p*  = 12 (see Fig. 1A) we took into account both the phenotypic effects of *q*_1_ = M←184 V, which arose earlier at passage 4, as well as *q*_2_ =  N←67 S, see eq. (1)-(3).

### Passage Times

**Figure 2:**
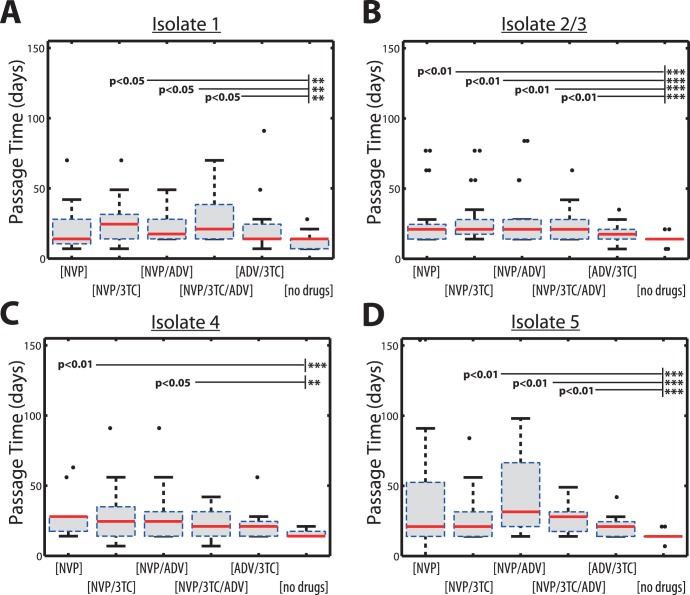
Box plot of single passage times for virus isolate #1, #2/3, #4 & #5 during experimental set-ups A–F as indicated on the x-axis. The solid red horizontal lines indicate the respective median passage times, whereas the boxes surrounding them indicate the range encompassed by the 25^th^ and 75^th^ percentiles. The whiskers denote the most extreme data points, which are not considered outliers and the black dots indicate outliers. A: Viral passage times for isolate #1. B: Viral passage times for isolate #2&3 (combined). C: Viral passage times for isolate #4. D: Viral passage times for isolate #5.

In the experiments (see *Virological Methods*), viral growth which exceeded a threshold 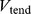 was recorded, i.e. the time required for a viral population to exceed a p24 ELISA signal of≥3×10^4^ pg/ml (see Fig. 2). This time can be referred to as *passage time*. Having a model for the growth of virus (see eqs.(1)-(3)), we can compute these quantities from the model as well, which allows us to perform parameter estimation. The passage experiments can be modelled as a Markov process [33], for which the time elapsed (in days) before the size of the virus population attains the threshold 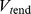 is referred to as *passage time* (in mathematical literature it is also referred to as *first passage time* or *first hitting time*) [34]. Because the *passage times* are random variables, we are interested in their statistical moments. The first statistical moment of the probability distribution of the *passage time* corresponds to its mean value (the *mean passage time*), whereas the square root of the second (centralized) statistical moment corresponds to its standard deviation [35].

In the passage experiments described above (see *Virological Methods*), virus was diluted 100-fold (100 µL supernatant in 10 mL media) and the time to an initial p24 ELISA signal (≥3×10^4^ pg/ml) was recorded. We therefore infer that 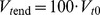; i.e. the concentration of virus has to increase by a factor of 100 with respect to the virus concentration used at the initiation of a passage 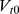. For any passage *p* during experimental setting *j* the *mean passage time* can be computed according to [36]:
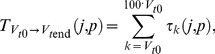
(4)where 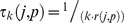 denotes the waiting time in state *k* (number of viral particles). Substituting eq.(1), we get 
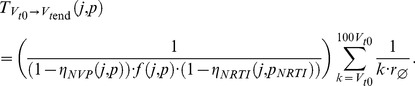
(5)


By further substituting eqs. (2)–(3), the equation above allows to express the *mean passage time* in terms of the 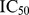, fitness values 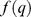, fold resistance 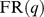, basic growth rate 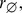 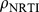 and 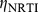, which will be exploited later for parameter estimation.

The *raw* second moment of the *passage time* distribution can be computed according to [36]:
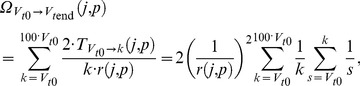
(6)where eq.(1)-(3) can again be substituted. The *raw* second moment (eq.(6)) can be centralized. The square root of this centralized second moment yields the *standard deviation* of the *passage times* [37]. Thus, the above analytical expressions enable to compute the *mean* 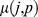 and *standard deviation* 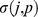 of the time required for a single-passage *p* in an experiment *j* according to 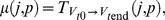
(7)

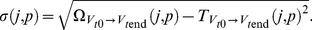
(8)


### Objective Function

The above derived *mean* 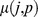 and *standard deviation* 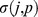 of the *passage times* correspond to a single passage *p* in an experiment *j*. Experimentally measured viral growth statistics (see Fig. 2) correspond to *mean* values and *standard deviations* pooled over all 12 individual passages *p* during experiment *j*. The corresponding pooled *means* 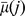 and *standard deviations* 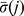 of *passage times* can be computed from the model as follows [38]:
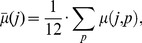
(9)

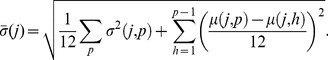
(10)


Substituting eqs.(1)-(8), we use the statistical measures derived in eqs.(9)-(10) to estimate model parameters (fold resistance towards NVP (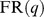) and the fitness deficits 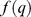 for single mutations *q*, the growth rate of the respective *baseline isolate* 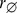, its susceptibility towards NVP (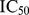) and the probability 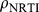 to encounter inhibition by NRTIs with intensity (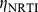), by minimizing the weighted least squares deviation between model and data:
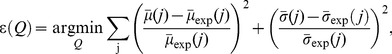
(11)were 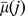 and 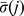 denotes the predicted *pooled mean passage times* and the corresponding *pooled standard deviations* respectively for experimental set-up *j*, which were computed using eqs.(9)-(10). Parameters 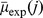 and 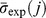 denote the experimental *pooled mean passage times* and their *standard deviations* for experimental set-up *j*, as shown in Fig. 2. The parameter set 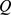, which is optimized, is determined by all mutational events that occurred in the respective isolate and baseline parameters. For example, for isolate #1, this includes the baseline parameters 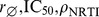 and 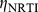 and the parameters related to mutational events: 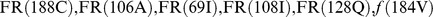


### Parameter Estimation, Identifiability & Model Selection

Parameter estimation was run using constrained optimization implemented in the MATLAB^©^ function ***lsqcurvefit*** (optimization toolbox). Note that some unbounded parameters (e.g. 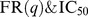) may not be reliably estimated if they appear in conjunction, see eq. (2). In order to improve the estimation of these parameters, we penalized unrealistically large values in the objective function, i.e. 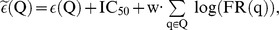 where 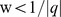. This way, a resistance value 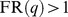 is only estimated, if it improves the model significantly over a ‘no resistance’ estimate 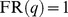 (‘null model’). Also, estimation would favor small 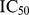 values, which is justified, because all baseline isolates were NVP-naive. While this change to the objective function adds a (small) bias towards lower 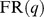 values, all fold resistance estimates have to be interpreted as lower boundaries, i.e. FR(106A)≥65 denotes that mutation V106A yields *at least* 65-fold susceptibility reduction. Parameter estimates for 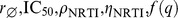 were not altered by this modification of the objective function.

Finally, we performed a model selection to investigate which sub-set of parameters 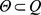 best explained the data. For example, if a total number of *two* mutational events *q*_1_ and *q*_2_ were selected in all experiments with the same isolate x, we took all of *four* possible candidate models 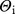 into account: a model that takes both mutational events *q*_1_ and *q*_2_ into account 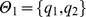, two models that take either *q*_1_ or *q*_2_ into account (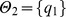 and 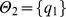) and a ‘null’ model 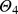, which does not take any mutational events into account. For each of the 2^|*q*|^ candidate models 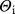 (in total 5280 models for all isolates), parameter estimation was performed 50 times with random start parameters to assess parameter identifiability for each candidate model 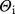. Finally, for each isolate, the *k*-most informative models according to their AIC (Akaike information criterion) [39] were chosen. The *k*-most informative models had to exceed a relative likelihood of 0.45, in comparison to the best model (lowest AIC). Parameter estimates presented later in the manuscript are medians and 5^th^ and 95^th^ percentiles based on the *k*-best models. A visual predictive check of experimental- vs. predicted data (*k*-best models) is shown in Figure S1. For clarity and ease-of-understanding, a diagram of the parameter estimation and model selection procedure is depicted in Figure S2 and the complete MATLAB^©^ source code is provided in Material S1, including a short instruction on its application.

## Results

### Selection of Mutations by ADV, 3 TC, and NVP

Baseline isolates #1–5 (see Table 1) exhibited thymidine-analogue-associated mutations (TAM): M41L, D67N, K70R, L210W, T215F/Y, K219Q and 3 TC resistance (M184V) [40], but there were no mutations associated with NNRTI resistance including positions 100,101, 103, 106, 108, 179, 181, 188, 190, 225, 227, 230 and 236 [28], [40], [41]. In experiments with NVP, substitutions at all of these positions were observed, except for codons 100, 225, 230 and 236. The additional substitutions observed at codons 69, 122, 128, 208, 218, 224 and 228 in our NVP experiments have not previously been linked to NVP resistance [28], [40]. Isolates exposed to escalating doses of NVP showed a gradual appearance/selection of one- to three new NNRTI mutations (totalling 43 new mutations with NVP), see Fig. 1. Of the newly detected mutations, 36 were at positions previously reported in the context of NVP resistance [28], [40], [41]. Mutations at position 106 (V106
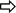
A/M/I) and V108
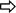
I were eventually selected by NVP (experimental set-ups 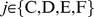). Interestingly, different substitutions arose for the distinct isolates at position 106, i.e. isolate #4 developed 106 M, whereas isolates #1 and #5 developed 106A and isolates #2/3 either developed 106A or 106I. The mutation V106
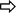
M always appeared with L228
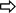
Q, followed by F227
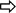
L in experiments with NVP and ADV (experiments D & F) in isolate #4. Mutations Y181
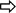
C and Y188
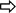
C were selected in isolates #2, 3, 4 & 5 and isolates #1, 2, 4 & 5 respectively. Other mutations occurred less frequently.

All isolates had the 184V mutation at baseline (Table 1), which was always preserved in the presence of 3 TC, whereas the reversion M←184V occurred in 86% (12/14) of the passages when 3 TC was absent (p<0.001). Interestingly, mutation H208Y was de-selected in isolate #4, experiments 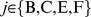, but selected in isolate #5, experiment 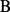, see Fig. 1B. Pre-existing TAM-1 mutations were deselected in isolate #4 C/D←215Y (Fig. 1A & Fig. 1C) and in isolate #5 L←210W (Fig. 1C & Fig. 1E). The mutation 210W was always deselected together with R←211K and in the presence of NVP, but in the absence of ADV (experiments C & E). The TAM-2 mutation at position 67 S was modified N←67 S in isolate #2/3, experiments 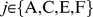.

Isolates #2 and #3 were derived from the same clinical isolate with similar, but not identical evolutionary patterns that emerged in serial passage experiments C, D & E (see Fig. 1C–E). This suggests that chance mutation may occur with different evolutionary consequences.

**Figure 3:**
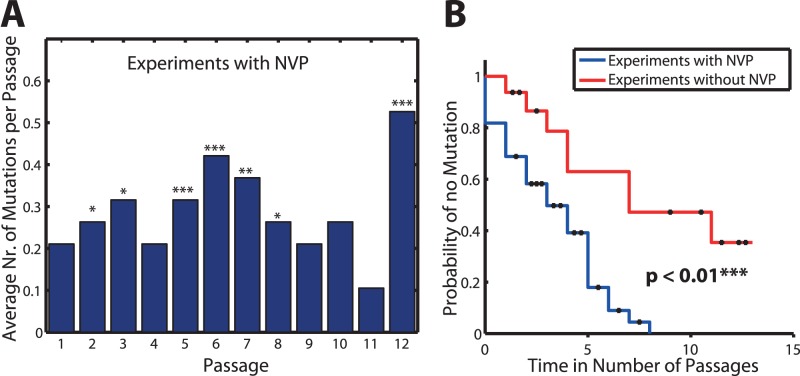
Selection dynamics. A: Average number of mutations per passage in experiments with NVP (experimental set-ups C, D, E & F). Asterisks indicate whether there were significantly more mutations (Wilcoxon rank sum test) than in the NVP-free experiments (experimental set-ups A & B). *p<0.1, **p<0.05, ***p<0.01. B: Cumulative probability of detecting no mutation. The blue and red lines show the cumulative probability of not detecting a mutation after the indicated numbers of passages (x-axis) in experiments where NVP was added with increasing concentrations (blue line; experimental set-ups C, D, E & F) vs. experiments where no NVP was added (red line; experimental set-ups A & B).

The average number of selected mutations is illustrated in Figure 3A for the sequential passages (pooling data from all passages and isolates). The average rate of resistance development in passages 5–7 and 12 was significantly greater in experiments with NVP than without NVP. The rate of new resistance mutations in the NVP experiments was highest during passages 5–7 and 12.

The rate of mutation as time-to-event, with NVP, was significantly greater than the rate of mutation without NVP. In the presence of NVP, at least one mutation occurred after 8 passages, whereas at least one mutation occurred in only 47% of all experiments without NVP by passage 8 (and in 66.6% of experiments without NVP after 12 passages). The cumulative probability that no mutation appears is shown in Figure 3B. The rate at which mutations appear is significantly higher (p<0.01) with NVP.

### Viral Growth Kinetics

The experimental single-passage times are shown in Figure 2 where the passage times with NVP at increasing concentrations were compared to those without NVP. Most experiments with NVP resulted in significantly longer passage times compared to those without addition of drugs (experimental set-up A; see solid horizontal bars in Figure 2). Remarkably, there was no significant difference in *mean passage times* between experiments with NVP (experimental conditions C, D, E & F) and experimental set-up B (ADV and 3 TC at constant drug concentrations).

### Addition of Low dose NRTIs Introduces Stochastic Viral Growth Dynamics

**Figure 4:**
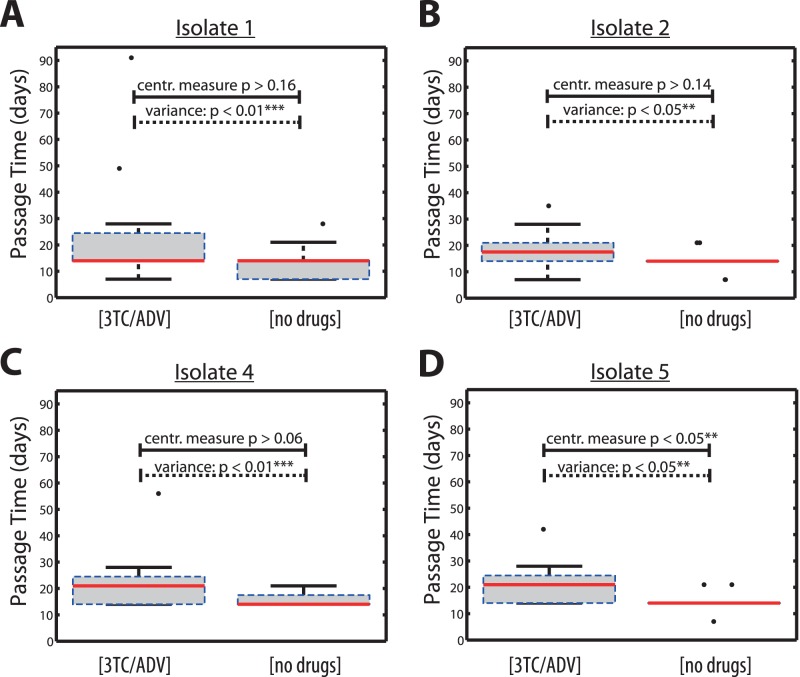
Box plot of passage times for virus isolate #1, #2, #4 & #5 during experimental set-ups A & B (no drugs added vs. 1 µM 3 TC plus 2 µM ADV added) as indicated on the x-axis. The solid red horizontal lines indicate the respective median passage times, whereas the boxes surrounding them indicate the range encompassed by the 25^th^ and 75^th^ percentiles. The whiskers denote the most extreme data points, which are not considered outliers and the black dots indicate outliers. A: Viral passage times for isolate #1. B: Viral passage times for isolate #2. C: Viral passage times for isolate #4. D: Viral passage times for isolate #5.

In Figure 4A–D we compared viral growth dynamics without drugs to growth in 1 µM 3 TC and 2 µM ADV. The addition of 3 TC and ADV did not significantly delay viral growth in isolates #1, 2 & 4, suggesting that 1 µM 3 TC and 2 µM ADV did not inhibit viral growth in the *majority* of passage-experiments. However, for isolate #5 significantly longer *passage times* were observed (p  = 0.01) when 3 TC and ADV were added to the medium. However, the *variance* of the *passage times* was significantly increased for all isolates tested (p<0.05 for isolates #2 and #5 and p<0.01 for isolates #1 and #4, see Fig. 4).

This initial evaluation of viral growth kinetics in the presence and absence of 3 TC/ADV indicates a stochastic effect of 3 TC and ADV. In order to account for this effect in the viral growth model, we introduced the parameter 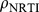 denoting the probability of NRTI-effect (depending on the baseline isolate), and a parameter describing the intensity of effect, denoted by 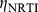. These parameters were estimated to be 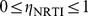 with probability 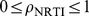 when NRTIs were added (experimental set-ups 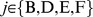) and set to 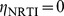, if NRTIs were not added (experimental set-up: 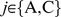), as described in the *Mathematical Methods* section.

### Estimating Drug Susceptibility and Fitness of Baseline Isolates

Using the *Mathematical Methods* described earlier, we were able to estimate key model parameters. In Table 2 the estimated growth rates *r*_∅_ and 50% inhibitory NVP concentrations IC_50_ are shown for the respective baseline isolates.

**Table 2:** Estimated baseline parameters.

	*r*_0/_[1/day]	IC_50_[µM]	*η*_NRTI_	*ρ*_NRTI_
**Iso #1**	0.42 (0.42, 0.42)	0.39 (0.37, 0.48)	0.65 (0.63, 0.67)	0.43 (0.28, 0.44)
**Iso #2/3**	0.39 (0.39, 0.39)	0.07 (0.07, 0.1)	0.99 (0.97, 0.99)	5.4e−7 (4.7e−7, 1.8e−4)
**Iso #4**	0.33 (0.33, 0.33)	0.13 (0.07, 0.14)	0.99 (0.99, 0.99)	1.1e−5 (3.8e−6, 1.2e−5)
**Iso #5**	0.36 (0.36, 0.36)	0.39 (0.39, 0.49)	0.99 (0.99, 0.99)	1e−6 (9.9e−7, 1.1e−6)

*r*_0/_ (baseline growth rate in the absence of drugs), intensity- *η*_NRTI_ and probability *ρ*_NRTI_ of NRTI-induced effect at concentrations of 1 µM 3 TC and 2 µM ADV respectively and lower-bound estimates for the susceptibility of baseline isolates towards NVP IC_50_. Indicated numbers are median estimates from the *k-*best models (see Mathematical Methods) and their respective 5^th^–and 95^th^ –percentiles (in brackets).

All baseline isolates had fairly similar growth rates (range: 0.33–0.42 day^−1^), although isolate 1 seems to be slightly more fit (in terms of the viral growth rate *r*_∅_), whereas isolate 4 is the least fit of the four baseline isolates. The estimated median IC_50_ of the baseline isolates ranged from 0.07 to 0.39 µM NVP, consistent with published IC_50_ values for drug susceptible virus (*wt*- IC_50_:0.1 µM [23]; corrected for protein binding).

The estimated intensity- and probability of NRTI effect (parameters *η*_NRTI_ and *ρ*_NRTI_ respectively) are shown in Table 2. For isolates #2, #3, #4 and #5, the estimated parameters confirmed that the probability of NRTI inhibition is low (*ρ*_NRTI_ = 5.10^–7^,10^–5^ and 10^–6^ respectively for isolates #2/3, #4 and #5), but the intensity of effect was quite pronounced (*η*_NRTI_ = 0.99 respectively for isolates #2/3, #4 and #5) at low NRTI concentrations. In contrast, for isolate #1, parameters relating to the efficacy of NRTIs were *ρ*_NRTI_ = 0.43 (5^th^ percentile: 0.28; 95^th^ percentile: 0.44) and *η*_NRTI_ = 0.66 (5^th^ percentile: 0.63; 95^th^ percentile: 0.67).

### NVP Drug Resistance

Using the *Mathematical Methods* presented earlier, we estimated the *fold resistance* FR(*q*), exerted by the individual mutations *q* (see Table 3). As described in the *Mathematical Methods* section, we used a model selection algorithm in order to choose the most informative models (permutations of considered mutations) for our parameter estimation. As a consequence, not all parameters could be estimated. Mutations that were observed during final passages did not allow the assessment of growth dynamics in subsequent passages. Specifically, parameters FR(69I), FR(103N), FR(122K), FR(128Q), FR(208Y) and FR(218G) were not identifiable from the data and were thus not included into Table 3.

**Table 3:** Estimated lower-bounds of fold resistance against NVP exerted by single amino acid substitutions in the distinct genetic background of the baseline isolates.

	Iso #1	Iso #2/3	Iso #4	Iso #5
**FR(101E)**	n.s	n.s	5 (2, 5)	n.s.
**FR(106A)**	80 (52, 135)	176 (22, 195)	n.s	21 (9, 47)
**FR(106I)**	n.s	5 (3, 9)	n.s	n.s
**FR(106M)**	n.s	n.s	1 (1, 4)	n.s
**FR(108I)**	25 (7, 26)	1 (1, 1)	7 (6, 65)	7 (3, 7)
**FR(179I)**	n.s.	1 (1, 3)	n.s.	n.s.
**FR(181C)**	5 (4, 6)	7 (6, 41)	n.i	13 (10, 13)
**FR(190A)**	n.s	8 (7, 11)	n.i	n.s.
**FR(181C/190A)**	n.s	n.s	67 (59, 300)	n.s.
**FR(188C)**	23 (4, 43)	n.s.	n.s	7 (2, 11)
**FR(218E)**	n.s.	1 (1, 5)	n.s.	n.s.
**FR(224K)**	n.s	1 (1, 5)	n.s	n.s.
**FR(227L)**	n.s	n.s	12 (7, 29)	n.i.
**FR(228Q)**	n.s	n.s	128 (8, 423)	n.s.

Values indicated are medians of all parameter estimates and the 5^th^ and 95^th^ percentile of the estimates are indicated in brackets. 'n.s’ means ‘not selected’ and n.i. means parameter ‘not identifiable’. Parameters FR(69I), FR(103N), FR(122K), FR(128Q), FR(208Y) and FR(218G) were not identifiable from the data for any isolates and thus omitted from the table.

Resistance estimates were distinct for the four different baseline isolates, indicating that pre-existing NRTI mutations may have influenced the impact of subsequent mutations on NVP susceptibility [42]–[45].

All isolates developed novel mutations at codon 106 in the presence of NVP. Mutation V106
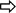
A was estimated to induce a profound fold resistance for isolates #1 and #2/3 and in isolate #4 (≥80, ≥184 and ≥21 respectively). Substitution V106
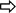
I was associated with at least 5-fold resistance, which was selected by relatively low NVP concentrations of 0.04 µM in isolates #2/3. According to our estimates (Table 3), the substitution V106
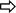
M in isolate #4 elicited little resistance.

Mutation V108
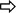
I arose in all isolates at least once. V108
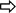
I led to modest NVP resistance in isolate #4 and #5, whereas moderate to strong resistance was conferred in isolate #1. Mutation Y181
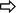
C appeared in all isolates, but the magnitude of resistance conferred by this mutation could only be estimated for isolates #1, #2/3 and #5, where it resulted in 5- to 13-fold resistance. In isolate #4, Y181**
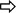
**C appeared simultaneously with G190???A, which induced strong NVP resistance according to our parameter estimates (FR≥67).

Interestingly, mutation L228
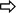
Q (isolate #4) was estimated to be associated with strong resistance development to NVP (Table 3) by our parameter estimates. This mutation always occurred before F227
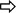
L, which added a moderate fold resistance. Mutation K103
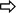
N, which is the most commonly observed resistance mutation to NVP in the clinic, appeared only during the final passage in experimental set-up D [NVP +3 TC] with isolate #2. Hence, a phenotype associated with this mutation was not observed and FR(103N) could not be estimated.

### Effect of Baseline Mutations on Viral Fitness

The following back mutations or reversals were observed during the passage experiments:

*q*∈{M←184V, N←67S, H←208Y, C/D←215Y, V←35I, L←210W, R←211K}.

Only the de-selection M←184V significantly improved viral fitness in all isolates according to our parameter estimates (Table 4). Interestingly, the number of distinct mutations undergoing reversal was inversely correlated with our estimates of the growth rate *r*_∅_, i.e. the “fittest” baseline isolates (largest parameter *r*_0/_) had the fewest distinct mutations reversing back to wild type (1, 2, 4, 2 distinct deselected mutations for isolates #1, #2/3, #4 and #5 respectively).

**Table 4:** Estimated relative fitness deficit *f(q)* of mutations present in the genetic background of baseline isolates #1,#2/3,#4,#5.

	Iso #1	Iso #2/3	Iso #4	Iso #5
***f*** **(184V)**	0.79 (0.79, 0.79)	0.59 (0.59, 0.62)	0.65 (0.64, 0.65)	0.65 (0.65, 0.66)
***f*** **(215Y)**	n.ds	n.ds	0.68 (0.68,0.68)	n.ds

A small value (close to 0) denotes a large fitness loss, whereas a value close to 1 denotes no fitness deficit. Values indicated are medians of all parameter estimates. The 5^th^ and 95^th^ percentile of estimates are indicated in brackets. 'n.ds’ means ‘not deselected’ and n.i. means parameter ‘not identifiable’. Parameters *f*(67S), *f*(208H), *f*(35I) and *f*(210W/211K) could not be reliably estimated from the data or were not significantly different from the value 1.

Parameter estimation (see Table 4) indicated that 184V conferred the greatest selective disadvantage of all mutations tested. The relative fitness estimated for the individual isolates ranged from 59% to 79% for the distinct isolates, which is generally consistent with previous *in vivo* estimates [46] and predictions from mechanistic mathematical models of HIV-1 DNA polymerization [7].

Although all baseline isolates carried resistance mutations at position 215, a change at amino acid position 215 (C←215Y and D←215Y) was observed only in isolate # 4. In Table 4, we estimated a relative fitness of 68% attributable to amino acid 215Y in isolate #4.

Fitness values *f*(67S), *f*(208H), *f*(35I) and *f*(210W/211K) were not included in the *k*-most informative models (see *Mathematical Methods*) and could thus not be estimated from the data.

## Discussion

The selection of drug resistance by a combination of drugs *in vitro* demonstrated complex evolutionary trajectories. Mathematical modelling of the passage experiments and the viral growth dynamics enabled estimation of fitness and drug resistance associated with mutational events.

### Epistasis and Combination Passage Experiments

Drug resistance emerging during antiretroviral combination therapy is influenced by viral genetic polymorphisms, random effects, and epistasis. The latter is a phenomenon where the phenotype induced by one mutation is modified by one or several other mutations [47]. Epistasis may be of particular clinical relevance in NRTI and NNRTI therapies, where the reverse transcriptase is targeted by different drugs [3]. Mutations resulting from exposure to one RT inhibitor may alter the phenotype of mutations selected by another RT inhibitor, through functional or conformational perturbation of the enzyme. Epistasis in HIV-1 has been studied in the absence of drugs [47] and after application of single drugs [7], [48]. Due to the complexity of the laboratory work required and the many possible permutations resulting from a variety of parameters, passage experiments using multiple drugs simultaneously have not yet been studied in detail. Here, combination passage experiments were performed in four clinical isolates from NRTI experienced, NNRTI-naive patients. In contrast to site directed mutagenesis to introduce resistance mutations into clonal laboratory isolates, clinical isolates were subject to the gradual and “natural” evolutionary dynamics in the serial passage experiments. Consensus sequencing after each passage allowed the detection of variants that may be favored by *in vivo* selection.

The isolates harbouring amino acid substitutions selected by NRTI exposure showed very distinct (strain-specific) evolutionary trajectories. Among identical amino acid substitutions we could observe distinct (strain-specific) effects on drug resistance (see Tables 2 & 3). Such strain-specific differences are likely governed by epistatic interactions between pre-existing and novel emerging mutations, as reported elsewhere [13], [14]. Epistasis in combination therapy complicates genotype-phenotype relationships because single mutations may have different effects on drug resistance in different genetic backgrounds.

The divergent evolution of isolates #2 and #3 (derived from the same baseline sample), on the other hand, stresses the impact of chance mutation on evolutionary trajectories.

### Phenotypic Attributes of Acquired NVP Resistance Mutations

Epistasis could be the mechanism behind the selective amino acid substitution at RT codon 106 in isolate #4, which exclusively developed the V106
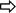
M (GTG -> **A**TG) substitution, although V106
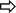
A (GTG -> GCG) could also have occurred by random mutation. Interestingly, V106
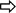
M always appeared together with L228
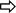
Q (either before- or after), followed by F227
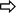
L in experiments with NVP and ADV (experiments D & F) in the specific genetic background of isolate #4. The L228
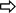
Q is a rare mutation associated with co-administration of NRTIs and NNRTIs [28], particularly when the NRTI is a dATP analogue such as ADV. In our case it only (and always) appears in isolate #4, i.e. in 2/2 experiments when NVP was co-administered with ADV (experiments D & F), irrespective of the presence of 3 TC. The L228
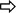
Q mutation results in a change from a non-polar/hydrophobic- to a strongly polar amino acid in direct proximity to the NNRTI binding pocket [49], possibly modulating the binding of NVP, which could induce resistance as predicted in Table 3.

In contrast, isolates #1, #2/3 and isolate #5 developed V106
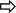
A, which mediate strong resistance [28] in agreement with our estimates, see Table 3. Although previous reports suggest that the V106
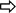
I mutation alone does not confer resistance to NVP *in vitro* [50], [51], we estimated a low-to-moderate resistance by this mutation, which may be explained by the genetic background of isolates #2/3.

It may be possible that appearance of the K103
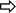
N mutation was restricted by multiple pre-existing RT mutations. Notably, the K103
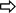
N may contribute little in terms of resistance to NVP in the presence of multiple TAMs and 3 TC resistance (such as RT mutations: M41L, D67N, M184V, L210W, T215Y, K219Q, P236P/L) [52], see Fig. 1.

Previous reports provide evidence that the Y181
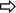
C mutation is associated with moderate-to-high level NVP resistance [28], while our estimates for isolates #1, #2/3 and #5 suggests a low-to-moderate resistance attributable to this mutation. Although considered a major resistance mutation against NVP, [28], [40], in isolate #3, the estimated impact of G190
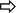
A was only moderate. In agreement with other data [28], [52], mutation Y188
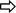
C induced moderate to strong NVP resistance. This mutation however, was only selected in isolates #1 and #5.

### Phenotypic Attributes of De-selected (Fitness) Mutations

The 184 V mutation was deselected in all combination passage experiments *not* including 3 TC and conferred a significant selective disadvantage in all isolates tested, which is exploited in some treatment lines that include 3 TC despite the M184V mutations. However, the degree of the fitness deficit was different for the four distinct baseline isolates, suggesting, again, epistatic interactions, as described previously [53], [54]. The statistical analysis of the experiments suggested that addition of 1 µM 3 TC and 2 µM ADV to the *drug resistant* viruses used in the experiments did not significantly inhibit viral growth. Furthermore, the analysis suggested that M184V, which was present in all baseline isolates, persisted in the presence of 1 µM 3 TC and reverted in its absence. The continued administration of 3 TC to preserve 184V is common in salvage strategies [18], [55].

Mutations C/D←215Y are considered “reversion” mutations that have been observed *in vivo* in the absence of zidovudine (AZT) [56], [57] and in untreated individuals infected with 215F/Y-containing (AZT-resistant) variants [57]. Here, “reversion” was observed in the genetic context of isolate #4. This “reversion” could recover an apparently large fitness loss associated with 215Y (relative fitness 68%, see Table 3). AZT-susceptible strains containing 215C/D were reported to be as fit as the WT virus in the absence of the drug [58], but retain the potential for the rapid emergence of high-level AZT resistance, through a single nucleotide substitution at codon 215 to become the resistant 215Y (by contrast, the conversion of wild type to resistant T215
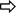
Y requires 2 nucleotide substitutions).

Multidrug passage experiments with escalating drug concentrations may reproduce clinical scenarios where concentrations vary over time and facilitate the development of drug resistance [59]. The emergence of drug resistance, despite high genetic barriers, has been attributed to heterogeneous pharmacokinetics in multiple physiologic compartments [60], resulting in sub-inhibitory concentrations of drugs [61]–[63]. In our passage experiments, such multi-stage scenarios can be reproduced: resistance may evolve under conditions allowing residual replication (the respective first passages) and then be selected further in subsequent passages favoring selection through increased selective pressure. The drug combination assay may be suitable to study the evolution of very complex resistance patterns, such as TAMs, the Q151M complex, resistance to some protease inhibitors or multidrug resistance to NNRTI+NRTI combinations.

Most importantly, *in vitro* combination passage experiments in conjunction with novel *in silico* analysis methodologies can contribute to an improved understanding of the complex evolution of drug resistance in clinical studies and individuals. Mathematical tools to estimate phenotypic parameters, including resistance and fitness, may provide new insights for designing effective drug combinations.

## Supporting Information

Figure S1Visual predictive checks of predicted (y-axis) versus observed (x-axis) data points. A: Means of mean first passage times 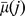
and B: their standard deviations 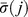. The distinct markers indicate the different patient isolates: leftward-, upward-, rightward- and downward-pointing triangles indicate data/predictions from/for isolates #1, #2/3, #4 and #5. Colours indicate the different experimental set-ups, e.g. red, cyan, blue, yellow, magenta and green denote experimental set-ups A-F respectively. Vertical bars indicate the range of predictions spanned by the 5^th^ and 95^th^ percentile of all model evaluations.(PDF)

Figure S2 Organization of the source code (Material S1) for the estimation of phenotypic parameters from passage experiments.
(PDF)

Material S1 MATLAB^©^ source code of the developed parameter inference procedure described in the *Mathematical Methods* section.
(ZIP)
