# Supplementary figures and images for: In Vitro HIV-1 Evolution in Response to Triple Reverse Transcriptase Inhibitors & In Silico Phenotypic Analysis
Source: PLoS One. 2013 Apr 17;8(4):e61102. doi: 10.1371/journal.pone.0061102 (PMC3629221; doi:10.1371/journal.pone.0061102)

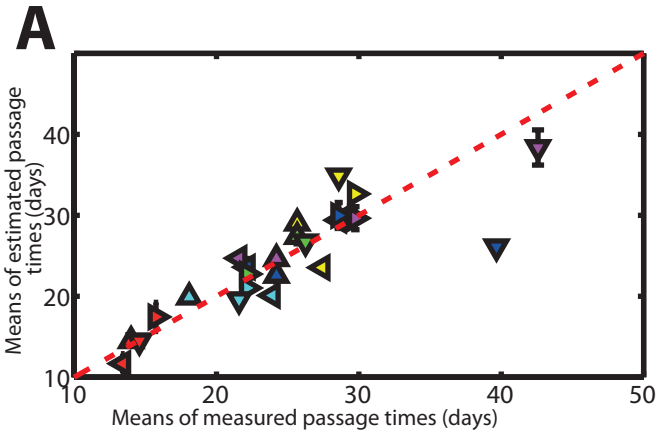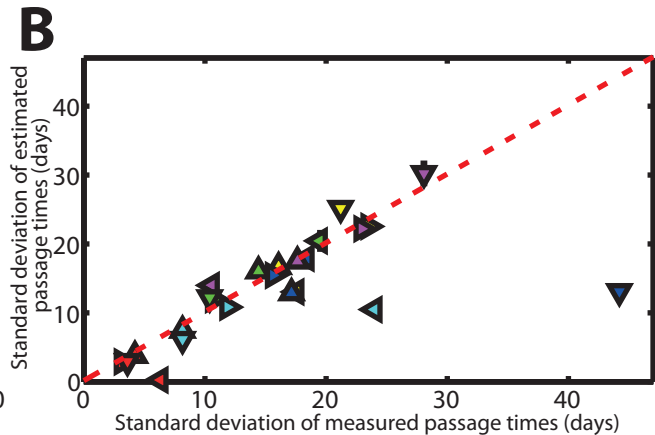

Supplement: Figure S1 — Visual predictive checks of predicted (y-axis) versus observed (x-axis) data points. A: Means of mean first passage times and B: their standard deviations . The distinct markers indicate the different patient isolates: leftward-, upward-, rightward- and downward-pointing triangles indicate data/predictions from/for isolates #1, #2/3, #4 and #5. Colours indicate the different experimental set-ups, e.g. red, cyan, blue, yellow, magenta and green denote experimental set-ups A-F respectively. Vertical bars indicate the range of predictions spanned by the 5th and 95th percentile of all model evaluations. (PDF) [file pone.0061102.s001.pdf]
