# Supplementary material for: In Vitro HIV-1 Evolution in Response to Triple Reverse Transcriptase Inhibitors & In Silico Phenotypic Analysis
Source: PLoS One. 2013 Apr 17;8(4):e61102. doi: 10.1371/journal.pone.0061102 (PMC3629221; doi:10.1371/journal.pone.0061102)
Supplement: Figure S2 — Organization of the source code (Material S1) for the estimation of phenotypic parameters from passage experiments. (PDF) [file pone.0061102.s002.pdf]

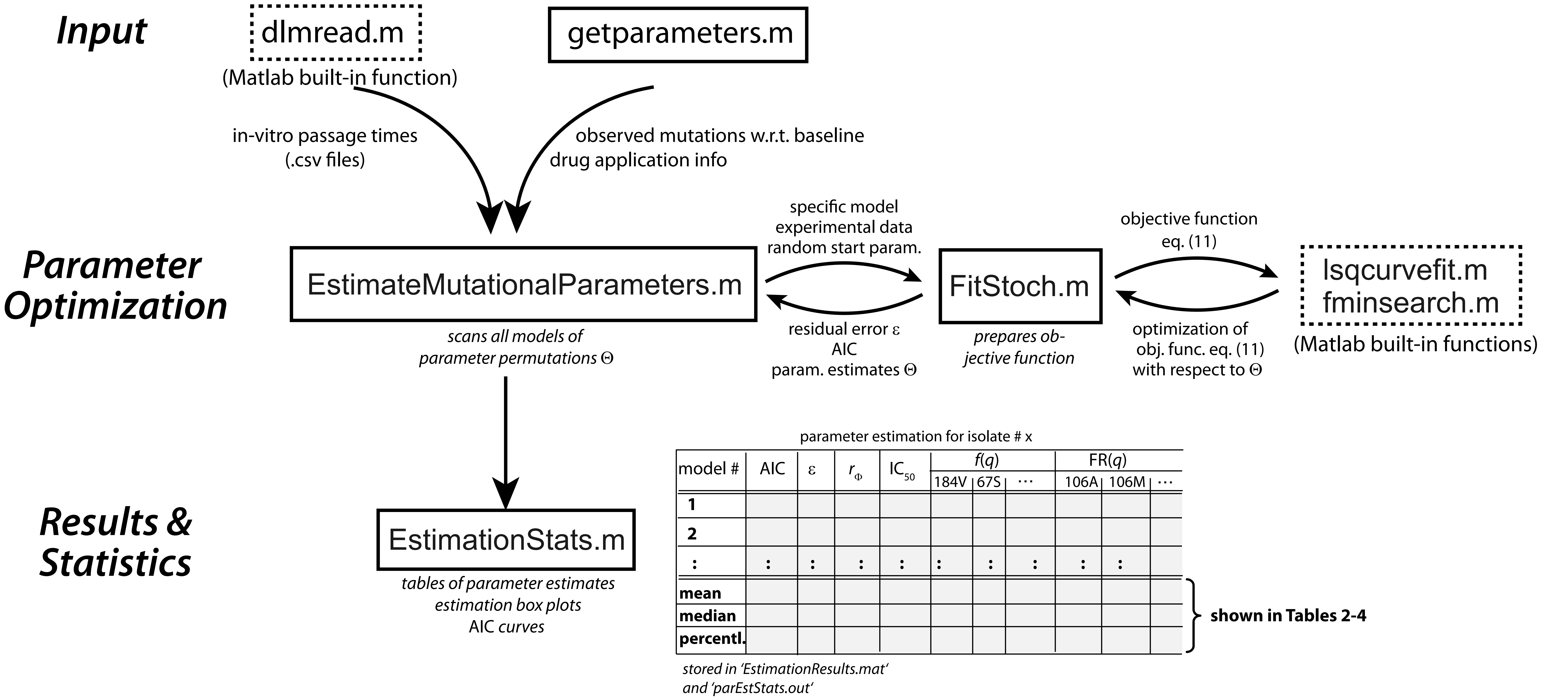

Input
dlmread.m
(Matlab built-in function)
getparameters.m
in-vitro passage times
(.csv files)
observed mutations w.r.t. baseline
drug application info
Parameter Optimization
EstimateMutationalParameters.m
scans all models of
parameter permutations Θ
specific model
experimental data
random start param.
residual error ε
AIC
param. estimates Θ
FitStoch.m
prepares objective function
objective function
eq. (11)
optimization of
obj. func. eq. (11)
with respect to Θ
lsqcurvefit.m
fminsearch.m
(Matlab built-in functions)
Results & Statistics
EstimationStats.m
tables of parameter estimates
estimation box plots
AIC curves
parameter estimation for isolate # x
model #
AIC
ε
r_Φ
IC_{50}
$f(q)$
184V
67S
FR(q)
106A
106M
1
2
mean
median
percentl.
shown in Tables 2-4
stored in 'EstimationResults.mat'
and 'parEstStats.out'
