# Supplementary material for: In Vitro HIV-1 Evolution in Response to Triple Reverse Transcriptase Inhibitors & In Silico Phenotypic Analysis
Source: PLoS One. 2013 Apr 17;8(4):e61102. doi: 10.1371/journal.pone.0061102 (PMC3629221; doi:10.1371/journal.pone.0061102)
Supplement: Material S1 — MATLAB© source code of the developed parameter inference procedure described in the Mathematical Methods section. (ZIP) [file pone.0061102.s003.zip › Application of the computational method.pdf]

Application of the computational method

The experimental data used for parameter estimation consisted of a) the mean duration of the passage experiments j $\bar{\mu}_{\mathrm{exp}}(j)$ and their standard deviation $\bar{\sigma}_{\mathrm{exp}}(j)$ (see Fig. 3) and b) the respective types of mutations and reversions q that occurred (see Fig. 1; these mutational events gave rise to the respective candidate models Θ_{i}).

In order to use the programmes provided in supplementary material S3, start "EstimateMutationalParameters.m" in MATLAB©. The program imports the experimental data (passage times, mutations) and generates candidate models Θ_{i} for parameter estimation. For each candidate model Θ_{i}, parameters are estimated 50-times using eq. (11) with random initial values. Estimated parameters and goodness-of-fit (in terms of the residual error $\varepsilon(\Theta_{\mathrm{i}})$) for each candidate model are written into an output file. The program "EstimationStats.m" can then be used in order to derive statistics for the estimated parameters.
